# Screening Colonoscopy Findings Are Associated With Noncolorectal Cancer Mortality

**DOI:** 10.14309/ctg.0000000000000479

**Published:** 2022-03-25

**Authors:** Brian A. Sullivan, Xuejun Qin, Cameron Miller, Elizabeth R. Hauser, Thomas S. Redding, Ziad F. Gellad, Ashton N. Madison, Laura W. Musselwhite, Jimmy T. Efird, Kellie J. Sims, Christina D. Williams, David Weiss, David Lieberman, Dawn Provenzale

**Affiliations:** 1 Cooperative Studies Program Epidemiology Center-Durham, Durham VA Health Care System, Durham, North Carolina, USA;; 2 Duke University, Durham, North Carolina, USA;; 3 Department of Solid Tumor Oncology, Levine Cancer Institute, Charlotte, North Carolina, USA;; 4 Perry Point VA Medical Center, Perry Point, Maryland, USA;; 5 VA Portland Health Care System, Portland, Oregon, USA;; 6 Oregon Health & Science University, Portland, Oregon, USA.

## Abstract

**INTRODUCTION:**

Controversy exists regarding the impact of various risk factors on noncolorectal cancer (CRC) mortality in healthy screening populations. We examined the impact of known CRC risk factors, including baseline colonoscopy findings, on non-CRC mortality in a screening population.

**METHODS:**

Cooperative Studies Program (CSP) #380 is comprised of 3,121 veterans aged 50–75 years who underwent screening colonoscopy from 1994 to 97 and were then followed for at least 10 years or until death. Hazard ratios (HRs) for risk factors on non-CRC mortality were estimated by multivariate Cox proportional hazards.

**RESULTS:**

Current smoking (HR 2.12, 95% confidence interval [CI] 1.78–2.52, compared with nonsmokers) and physical activity (HR 0.89, 95% CI 0.84–0.93) were the modifiable factors most associated with non-CRC mortality in CSP#380. In addition, compared with no neoplasia at baseline colonoscopy, non-CRC mortality was higher in participants with ≥3 small adenomas (HR 1.43, 95% CI 1.06–1.94), advanced adenomas (HR 1.32, 95% CI 0.99–1.75), and CRC (HR 2.95, 95% CI 0.98–8.85). Those with 1–2 small adenomas were not at increased risk for non-CRC mortality (HR 1.15, 95% CI 0.94–1.4).

**DISCUSSION:**

In a CRC screening population, known modifiable risk factors were significantly associated with 10-year non-CRC mortality. Furthermore, those who died from non-CRC causes within 10 years were more likely to have had high-risk findings at baseline colonoscopy. These results suggest that advanced colonoscopy findings may be a risk marker of poor health outcomes. Integrated efforts are needed to motivate healthy lifestyle changes during CRC screening, particularly in those with high-risk colonoscopy findings and unaddressed risk factors.

## INTRODUCTION

Most individuals undergoing routine colorectal cancer (CRC) screening and surveillance will die from causes other than CRC (1). Given that these individuals have demonstrated adherence and a desire for routine preventative health care, CRC screening is a unique opportunity to augment efforts to help also prevent non–CRC-related poor health outcomes (2–7). Gastroenterologists could play a role in the overall health of patients, beyond CRC prevention, by providing brief counseling or referrals for any identified factors that may be detrimental to health such as poor diet, inadequate exercise, tobacco use, or other issues (e.g., medication adherence, sleep apnea, mental health, or dental care) (8).

Yet despite the well-known associations of many diet and lifestyle factors for premature mortality (9), studies investigating the impact of participating in CRC screening on modifying these risk factors have been inconclusive (3,4). Diet and lifestyle factors may be going unaddressed or in some cases could worsen after CRC screening (5). This is especially true since controversy exists regarding which clinical and lifestyle factors most impact mortality risk. Clarification of the strength of association between known risk factors and non-CRC mortality, specifically in healthy screening populations, is an unmet need that could improve health outcomes by leading to the development of personalized strategies integrated with CRC screening that efficiently prioritize healthy lifestyle interventions most relevant to the individual.

Individuals undergoing CRC screening represent an important, yet challenging, population to investigate non-CRC mortality risk factors. As they are typically healthy overall, it is often difficult to accurately identify the subset of individuals most at risk for poor outcomes and thus more likely to benefit from aggressive risk factor modification. Effective markers for identifying individuals at risk for short-term (i.e., 10-year) non-CRC mortality in healthy CRC screening populations remain elusive, but could lead to improved efforts at risk factor modification. Recent data now suggest that advanced findings on colonoscopy could be linked to underlying factors that increase mortality. In fact, screening colonoscopy findings themselves may represent a readily available novel marker for characterizing underlying health risk (6,10,11). In other words, initial colonoscopy findings may highlight the immediate real-world consequences of (previously uninvestigated) unhealthy lifestyle choices, inform assessments of short-term non-CRC mortality, and motivate clinical decision making regarding optimal health strategies. Indeed, if screening colonoscopy findings are shown to be associated with poor non-CRC health outcomes, individuals found to have clinically significant lesions on initial colonoscopy may be those most likely to benefit from concerted lifestyle interventions that decrease non-CRC mortality risk (7).

This study investigated a prospective screening colonoscopy cohort to examine the strength of association with non-CRC mortality for a focused set of baseline demographic, clinical, and lifestyle factors. This study also evaluated whether baseline colonoscopy findings were associated with non-CRC mortality in this healthy population.

## METHODS

Cooperative Studies Program (CSP) #380 is comprised of 3,121 healthy veterans aged 50–75 years who underwent screening colonoscopy from 1994 to 97 (12). This analysis was approved by the Durham VA Medical Center Institutional Review Board (MIRB #1872) and included a waiver of informed consent for work performed under this protocol. All methods were performed in accordance with relevant guidelines and regulations.

Since the baseline screening colonoscopy, the clinical trajectories of these participants have now been well characterized through at least 10 years or until death. Details of this cohort, including measurement of baseline characteristics and 10-year clinical outcomes, have previously been described (13–15). At the initial screening colonoscopy, baseline findings were categorized as no neoplasia, 1–2 small (<10 mm) adenomas, 3+ small adenomas, advanced adenomas (defined as adenoma ≥10 mm, or with villous histology or high-grade dysplasia), or invasive CRC. Race groups (based on self-report in the baseline questionnaire) were defined as White, African American, and other (including Hispanic, Asian, and not otherwise specified). Baseline health status was estimated by self-reported conditions and verified in the electronic medical record. We categorized the number of health conditions into 0–2, 3–4, or 5+ conditions as a general measure of baseline health status and included diagnoses of cardiovascular disease, diabetes, and/or other cancers. Body mass index (BMI) was calculated as weight in kilograms divided by the square of height in meters. Information on family history of CRC, diet, physical activity, and alcohol, smoking (including current and past habits), and medication use was also obtained from validated self-reported questionnaires. In brief, diet was assessed with a validated semiquantitative food frequency questionnaire (Table 4). The physical activity index generated a 24-hour score based on patients reported activity levels. Categories of smoking, alcohol, and nonsteroidal anti-inflammatory drug (NSAID) use were defined based on detailed queries of cigarette habits, number of drinks per week, and specific NSAID products, respectively. Additional details are available at https://www.research.va.gov/programs/csp/cspec/datadictionary_csp380.html.

Cause of death information was obtained from the CSP#380 database, National Death Index, and the VA Corporate Data Warehouse. Specifically, CRC-specific mortality was confirmed in the CSP#380 database by a previously validated method using National Death Index cause of death codes cross-referenced with CRC diagnosis codes from the VA Corporate Data Warehouse (16). Few discrepancies were adjudicated by the CSP#380 team, similar to previous studies (17). Participants with CRC-specific mortality were removed to create the non-CRC mortality analytic data set.

Descriptive statistics, including rates and proportions for categorical data and means and SEs for continuous data, were used to describe baseline risk factors for the entire CSP#380 cohort. After excluding instances of CRC death, hazard ratios (HRs) and 95% confidence intervals (CIs) for baseline colonoscopy findings and other risk factors on non-CRC mortality were estimated by a Cox proportional hazards model, which adjusted for *a priori* selected demographics, baseline comorbidities, lifestyle factors, and colonoscopy surveillance intensity based on the initial CSP#380 randomization protocol. Finally, in a robustness analysis, we evaluated the same baseline risk factor associations with all-cause mortality, i.e., including CRC mortality in the full CSP#380 cohort. Study database management was performed using SAS, version 6.4 (SAS Institute, Cary, NC). All other analyses were performed using R, version 4.0.3, with the survival package. For all analyses, *P* < 0.05 was considered statistically significant.

## RESULTS

Participants were mostly male (n = 3,021, 96.8%) and White (n = 2,609, 83.6%) with a mean age of 62.9 years at baseline (Table 1). During the 10-year follow-up period, 858 (27.5%) died, including 843 (98.3%) from non-CRC causes (Table 2). These deaths were approximately evenly split between time periods of 0–5 years after baseline colonoscopy (42.5%) and >5–10 years from baseline (57.5%). Few deaths were attributable to CRC (n = 15, 0.5%), and most were within 5 years of the baseline examination (86.7%). Table 3 shows the primary cause of non-CRC mortality for CSP #380 participants during the 10-year follow-up period. Most deaths were related to cardiovascular disease (n = 324, 38.4%) followed by non-CRC malignant neoplasms (n = 207, 24.6%) and respiratory diseases (n = 78, 9.3%), which is consistent with US national cause of death statistics in this age group (1).

**Table 1.** Baseline characteristics of CSP #380

Characteristic	Participants (n = 3,121)
Age, mean (±SE)	62.9 (0.13)
50–59 yr, n (%)	1,044 (33.5)
60–69 yr, n (%)	1,481 (47.5)
>69 yr, n (%)	596 (19.1)
Male sex, n (%)	3,021 (96.8)
Race, n (%)	
White, non-Hispanic	2,609 (83.6)
Black, non-Hispanic	297 (9.5)
Other	215 (6.9)
Daily smoker, n (%)	691 (22.1)
Family history of CRC, n (%)	434 (13.9)
Any follow-up colonoscopy, n (%)	1,915 (61.4)

CRC, colorectal cancer; CSP, Cooperative Studies Program.

**Table 2.** Cause of death and timeline for CSP #380 participants

Deceased 10.5-yr	All CSP #380 participants (n = 3,121)	Deceased between 0 and 5 yr from baseline	Deceased between >5 and 10 yr from baseline
Total deaths, n (%)	858 (27.5)	365 (42.5)	493 (57.5)
CRC, n (%)	15 (0.5)	13 (86.7)	2 (13.3)
Other death, n (%)	843 (27.0)	352 (41.8)	491 (58.2)

CRC, colorectal cancer; CSP, Cooperative Studies Program.

**Table 3.** Primary cause of death for CSP #380 participants, excluding CRC

Primary cause of death	No. of deaths (n = 843)
Cardiovascular diseases, n (%)	324 (38.4)
Malignant neoplasms (non-CRC), n (%)	207 (24.6)
Respiratory diseases, n (%)	78 (9.3)
Endocrine, nutritional, and metabolic diseases, n (%)	51 (6.0)
Infectious and parasitic diseases, n (%)	49 (5.8)
Accidents (unintentional injuries), n (%)	41 (4.9)
Disease of the nervous system, n (%)	29 (3.4)
Gastrointestinal diseases, n (%)	22 (2.6)
Genitourinary tract diseases, n (%)	8 (0.9)
Intentional self-harm (suicide), n (%)	6 (0.7)
All other causes/unknown, n (%)	28 (3.3)

CRC, colorectal cancer; CSP, Cooperative Studies Program.

Table 4 demonstrates the impact of baseline characteristics on non-CRC mortality. Non-CRC mortality was higher in African American individuals (HR 1.26, 95% CI 0.99–1.62). Increasing age at baseline was significantly associated with higher non-CRC mortality (HR 1.07 per year, 95% CI 1.06–1.09) in the CSP#380 cohort. Non-CRC mortality was also higher in those with 3–4 medical comorbidities at baseline (HR 1.57, 95% CI 1.30–1.88, compared with 0–2 points) and in current smokers (HR 2.12, 95% CI 1.78–2.52, compared with nonsmokers). On the other hand, reduced non-CRC mortality was associated with increasing physical activity (HR 0.89, 95% CI 0.84–0.93) and a family history of CRC (HR 0.76, 95% CI 0.60–0.96). Increased BMI of 24.9–29.9 kg/m^2^ (HR 0.73, 95% CI 0.59–0.89) and 29.9–39.9 kg/m^2^ (HR 0.75, 95% CI 0.60–0.93) was also associated with decreased non-CRC mortality when compared with a BMI of 18.5–24.9 kg/m^2^ at baseline. Finally, neither NSAID use (including aspirin) nor dietary factors were associated with non-CRC mortality.

**Table 4.** Impact of baseline characteristics on non-CRC mortality in the CSP#380 cohort^a^

Baseline characteristic	Total (n = 3,106)	Deaths (n = 834)	Unadjusted hazard ratio (95% CI)	Adjusted hazard ratio^b^ (95% CI)
Age, yr, mean (SD)	62.9	65.37	1.06 (1.05–1.07)	1.07 (1.06–1.09)
Race, n (%)				
White	2,593	703 (27.1)		Ref
African American	296	85 (28.7)	1.08 (0.86–1.35)	1.26 (0.99–1.62)
Other	217	55 (25.3)	0.93 (0.71–1.23)	1.02 (0.74–1.39)
Baseline colonoscopy findings, n (%)				
No adenomas	1,948	481 (24.7)		Ref
1–2 small adenomas	683	195 (28.5)	1.17 (0.99–1.38)	1.15 (0.94–1.4)
3+ small adenomas	157	60 (38.2)	1.61 (1.23–2.11)	1.43 (1.06–1.94)
Advanced adenoma	298	101 (33.9)	1.46 (1.18–1.82)	1.32 (0.99–1.75)
Colorectal cancer	20	6 (30)	1.26 (0.56–2.84)	2.95 (0.98–8.85)
Baseline comorbidities, n (%)				
0–2	2,627	632 (75.0)		Ref
3–4	441	189 (22.4)	2.02 (1.71–2.37)	1.57 (1.30–1.88)
5+	38	22 (2.6)	3.23 (2.11–4.94)	1.65 (0.94–2.91)
Family history of CRC, n (%)				
First degree relative with CRC	432	95 (22.0)	0.76 (0.61–0.94)	0.76 (0.60–0.96)
Baseline body mass index, kg/m^2^				
18.5–24.9	457	155 (33.9)		Ref
<18.5	14	8 (57.1)	1.84 (0.91–3.75)	1.32 (0.64–2.72)
25.0–29.9	1,351	344 (25.4)	0.69 (0.57–0.84)	0.73 (0.59–0.89)
30.0–39.9	1,135	295 (26.0)	0.70 (0.58–0.86)	0.75 (0.60–0.93)
>39.9	129	34 (26.4)	0.73 (0.50–1.06)	1.05 (0.70–1.57)
Baseline physical activity index				
Mean score (SD)	7.16 (1.7)	6.81 (1.55)	0.84 (0.80–0.88)	0.89 (0.84–0.93)
Baseline smoking, n (%)				
Current daily smoker	691	257 (30.6)	1.70 (1.47–1.97)	2.12 (1.78–2.52)
Baseline alcohol use				
Mean (SD) servings per week	4.45 (9.54)	4.36 (10.74)	1.00 (0.99–1.01)	1.00 (0.99–1.01)
Baseline NSAID use (including aspirin), n (%)				
None	606	152 (25.1)		Ref
Occasional	756	163 (21.6)	0.84 (0.68–1.05)	0.97 (0.76–1.23)
Daily	1,654	502 (30.4)	1.24 (1.04–1.50)	1.2 (0.98–1.46)
Baseline beef, pork, or lamb consumption				
Mean (SD) % of total energy intake	0.97 (0.59)	1 (0.59)	1.09 (0.97–1.22)	1.04 (0.92–1.19)
Baseline cereal fiber consumption				
Mean (SD) grams	5.68 (3.94)	5.67 (3.85)	1.00 (0.98–1.02)	1.00 (0.98–1.02)
Vitamin D				
Mean (SD) in 100 IU units	4.28 (3.27)	4.32 (3.49)	1.01 (0.98–1.03)	0.99 (0.97–1.02)

CI, confidence interval; CRC, colorectal cancer; CSP, Cooperative Studies Program; NSAID, nonsteroidal anti-inflammatory drug.

a Missing data excluded.

b Adjusted for age, race, surveillance intensity and previous colonoscopy findings, number of baseline comorbidities, family history of CRC, smoking status, alcohol consumption, physical activity index, NSAIDs, body mass index, cereal fiber, red meat, and vitamin D.

Compared with no neoplasia at baseline colonoscopy, 10-year non-CRC mortality was higher in participants with ≥3 small adenomas (HR 1.43, 95% CI 1.06–1.94), advanced adenoma (HR 1.32, 95% CI 0.99–1.75), and CRC (HR 2.95, 95% CI 0.98–8.85) (Table 4). Those with 1–2 small adenomas at baseline were not at increased risk for non-CRC mortality (HR 1.15, 95% CI 0.94–1.4).

Supplementary Table 1 (see Supplementary Digital Content 1, http://links.lww.com/CTG/A784) demonstrates the results of a robustness analysis evaluating the impact of the same baseline risk factors on the outcome of all-cause mortality (which includes CRC-specific mortality). Qualitatively similar results to the analyses with non-CRC mortality were found, including similar strengths of association (i.e., magnitude of relative risk) across all endoscopic, clinical, and lifestyle baseline risk factors.

## DISCUSSION

In a healthy CRC screening population, we found that increasing age, African American race, more medical comorbidities, and current smoking were associated with higher rates of 10-year non-CRC mortality, whereas increased BMI, higher physical activity, and a family history of CRC were protective of short-term non-CRC mortality. In addition, high-risk findings (≥3 small adenomas, advanced adenoma, and CRC) on baseline colonoscopy are likely to be valuable predictors of increased non-CRC mortality risk within 10 years. To our knowledge, this is the first study to report an association between baseline colonoscopy findings and non-CRC mortality in a prospective screening population.

Several potentially modifiable risk factors for non-CRC mortality were identified in this healthy CRC screening population, including increased number of medical comorbidities, smoking, and decreased physical activity. Previous studies in various populations have found consistent relationships between all-cause mortality and these risk factors, all with similar effect sizes to our study (11,18–29). Although these findings are not novel, several specific findings warrant additional consideration. We observed similar associations between non-CRC mortality and African American race as has been reported in other VA and non-VA populations, although this disparity may be partially mitigated in the VA equal-access health system (30,31). Our finding that a BMI range from 25 to 39.9 kg/m^2^ is protective in terms of non-CRC mortality, when compared with a healthy BMI range of 18.5–24.9 kg/m^2^, was initially surprising. However, a “u-shaped” mortality curve of BMI has been well described in 2 large systematic reviews of heterogeneous populations over short follow-up periods (19,29). A family history of CRC was also associated with reduced non-CRC mortality (HR 0.75). The reasons are not known, but these individuals may generally be more health conscious given an awareness of familial risk (32). Further study is needed to explore this hypothesis. On the other hand, besides smoking (HR >2), the risk factor with the highest impact on non-CRC mortality in our analysis was increasing number of self-reported comorbidities at baseline. This finding is expected, but also reinforces the importance of strongly considering comorbidities in risk/benefit assessment of ongoing CRC surveillance (rather than just age alone) (23). In regard to aspirin use, we anticipated some impact on non-CRC mortality based on cardiovascular protection. Although 1 study by Loomans-Kropp et al. (33) showed a strong impact of aspirin on reduced all-cause mortality, a previous systematic review by the USPTF found that daily aspirin use of ≥75 mg was associated with only a small reduction (HR 0.94, with upper CI 0.99) in all-cause mortality within 10 years (34). Our study is unable to elucidate any potential benefits of aspirin given the small sample size and the inclusion of NSAID users beyond aspirin. Finally, we found no dietary factors associated with non-CRC mortality. Previous evidence in other studies demonstrates that unprocessed red meat may only have a very small risk reduction (with very low certainty evidence) (35,36), data on cereal fiber are mixed (37–39), and a recent a meta-analysis by Zhang et al. (40) found a lack of association between vitamin D supplementation and all-cause mortality. However, dietary studies are difficult to conduct given the subjective approximation that goes into self-questionnaires, as well as the imprecise measurement of total intake, duration, and various dietary interactions.

In addition, we found that non-CRC mortality was also higher in participants with advanced findings on baseline colonoscopy. When compared with those with no neoplasia on screening colonoscopy, the risk for non-CRC mortality increased by severity of findings, with point estimates of approximately 1.3 times in those with advanced adenomas to almost 3 times the risk if CRC was identified at baseline, even after adjusting for other important health risks such as smoking and comorbidities (41). Although previous studies have not typically reported outcomes of non-CRC mortality, a few have reported data suggesting a utility of colonoscopy findings for all-cause mortality risk prediction during CRC screening and surveillance. A study by Loberg et al. (42) found that individuals classified as having either low-risk (SMR 1.19; 95% CI 1.16–1.22) or high-risk adenomas (SMR 1.20; 95% CI 1.18–1.23) had higher all-cause mortality than the general population. Given that these effect sizes are similar to our study (see Supplemental Table 1, Supplementary Digital Content 1, http://links.lww.com/CTG/A784), it is therefore likely that the estimates from our study are a plausible assessment of the association between baseline colonoscopy findings with non-CRC mortality. In fact, emerging data now suggest that increased short-term mortality risks in CRC survivors may be attributable to shared underlying risk factors for both CRC development and non-CRC mortality, including lifestyle or metabolic risk factors that increase the risk for cardiovascular disease and other non-CRC malignancies relative to the general population (11,43–45). Extending this relationship of shared underlying risk factors for other poor health outcomes and precancerous advanced adenomas or multiple adenomas could explain the results of our study. Further, this supports the suggestion that more advanced colonoscopy findings may be a general marker for underlying risk factors related to poor health that have not yet been identified or targeted during routine health maintenance.

Although screening colonoscopy is a distinct touch point with the health care system for those interested in CRC prevention, it also represents an ideal opportunity to identify and mitigate risk for non-CRC mortality. Previous studies have evaluated whether known health risk factors change after CRC screening, but the data are mixed. The Norwegian Colorectal Cancer Prevention sigmoidoscopy study reported worsened dietary choices with increased weight, reduced physical activity, and more smoking after screening (5). On the other hand, Knudsen et al. (7) found that after screening, adherence to a healthier lifestyle modestly increased, but with more improvement in those with more advanced findings. Taken together with our results, these findings by Knudsen et al. suggest that those with more advanced findings at initial colonoscopy could be ideal targets for aggressive risk factor modification efforts. Advanced colonoscopy findings could represent a canary in the coal-mine scenario and allow for better identification of those most at risk for poor health outcomes and thus most likely to make and benefit from durable healthy changes. And because the associations in our study persisted over a 10-year follow-up period, it is likely that these factors were inadequately managed during routine health care maintenance. Therefore, by providing objective data (i.e., high-risk colonoscopy findings) that quantify risk in a unique way, postprocedure counseling is an opportunity for gastroenterologists to partner with a multidisciplinary team who continuously engage, reassess, and help patients manage risk or make shared decisions about lifestyle over time. In addition to readily apparent recommendations such as regular exercise, healthy diet, and smoking cessation (highlighted in our study), important factors that may also be actionable include hydration, dental care, sleep hygiene, mental health, comorbidity management (including medication adherence), and regular health maintenance visits and screenings. Even incremental changes, motivated by multiple providers, may add up overtime to improved outcomes (8,22). Given that participants undergoing CRC screening and surveillance rarely die from CRC, our findings provide more support for integrating efforts with CRC screening that seek to improve overall health by personalizing approaches to health maintenance, individualizing risk assessments, and effectively using targeted health interventions for relevant risk factor modification.

This study has limitations. Although the associations of African American race, baseline advanced adenoma, or CRC with non-CRC mortality did not meet the traditional dichotomous threshold for statistical significance (which may have occurred in a larger study), the point estimates and confidence intervals are most compatible with clinically relevant associations with non-CRC mortality and are likely to be valuable predictors of a poor health outcomes (41). These findings are consistent with other studies and ideas as described above, and at a minimum, suggest that these findings be a moment to assess for the presence of, and potentially help modify, important health risk factors. Otherwise, this is a veteran cohort of mostly White men aged 50–75 years, and so generalizability to other groups may be limited. In addition, baseline data, particularly regarding lifestyle factors, medication use, and family history of CRC were obtained by self-reported questionnaires, which may be subject to reporting or other bias. We also do not report changes in these risk factors over time, so we are unable to determine the impact of modifying these risk factors on specific causes of death. This will be an important focus of future research. All of the participants received screening; thus, we are unable to estimate the impact of various screening findings on non-CRC mortality compared with an unscreened population or those undergoing CRC screening with noninvasive modalities. Finally, because we did not analyze cause-specific outcomes of death individually, future work will seek to better understand the factors that most contributed to specific causes of short-term mortality risk in this cohort. Investigations of cause-specific mortality patterns will be important to develop potential high-yield strategies for risk modification during CRC screening and surveillance and/or determine when competing risks of comorbidities outweigh benefit of CRC screening and surveillance (46).

In conclusion, we found several known modifiable risk factors in a CRC screening population that are significantly associated with 10-year non-CRC mortality. Furthermore, those who died from non-CRC causes within 10 years were more likely to have had high-risk findings at baseline colonoscopy (≥3 small adenomas, advanced adenoma, and CRC), even after accounting for other important risk factors such as advanced age, increased comorbidities, and smoking. These results suggest that advanced colonoscopy findings may be an early marker for underlying risk of poor health outcomes and support integrated efforts with CRC screening to motivate healthy lifestyle change in these individuals. Future work will seek to understand whether a better understanding of individualized short-term mortality risk calculated at screening colonoscopy could inform prioritization of actionable risk reduction strategies and improve overall health outcomes.

## CONFLICTS OF INTEREST

**Guarantor of the article:** Brian A. Sullivan, MD, MHS.

**Specific author contributions:** B.A.S., D.P., E.R.H., and Z.F.G.: study concept and design. B.A.S., X.Q., T.S.R., L.M., K.J.S., and A.M.: acquisition of data. B.A.S., X.Q., C.D.M., K.J.S., T.S.R., E.R.H., and D.P.: analysis and interpretation of data. B.S., E.R.H., Z.F.G., and D.P.: drafting of the manuscript. J.T.E., K.J.S., C.D.W., L.W.M., D.W., and D.L.: critical revision of the manuscript for important intellectual content. X.Q., C.M., T.S.R., and E.R.H.: statistical analysis. E.R.H., D.L., and D.P.: obtained funding. A.M.: administrative, technical, or material support. E.R.H., D.L., and D.P.: study supervision.

**Financial support:** This work was funded by the US Department of Veteran Affairs Cooperative Studies Program.

**Potential competing interests:** B.A.S. reports grant support from Exact Sciences, which is outside the submitted work. D.L. reports other support from Check-Cap, Ironwood, ColoWrap, and Freenome, which is outside the submitted work. No other authors have anything to disclose. The views expressed in this article are those of the authors and do not necessarily represent the views of the Department of Veterans Affairs or the government of the United States.

**Data availability:** Analysis code is available on reasonable request (https://www.vacsp.research.va.gov/CSPEC/Studies/INVESTD-R/CSP-380-Risk-Factor-Colonic-Adenomas.asp).

**Study Highlights**

**WHAT IS KNOWN**

✓ Colorectal cancer (CRC) screening is an opportunity to address many important health factors.
✓ However, controversy exists regarding the impact of various risk factors in healthy screening populations.
✓ Furthermore, whether screening colonoscopy findings are also associated with non-CRC mortality is unknown.

**WHAT IS NEW HERE**

✓ High-risk findings on baseline screening colonoscopy are associated with non-CRC mortality within 10 years.
✓ Advanced colonoscopy findings could help identify those with unaddressed risks for poor health outcomes.
✓ Integrated efforts at screening colonoscopy are needed to help motivate healthy lifestyle changes.

**Supplementary Material**

SUPPLEMENTARY MATERIAL
