# Supplementary figures and images for: Screening Colonoscopy Findings Are Associated With Noncolorectal Cancer Mortality
Source: Clin Transl Gastroenterol. 2022 Mar 25;13(4):e00479. doi: 10.14309/ctg.0000000000000479 (PMC9038496; doi:10.14309/ctg.0000000000000479)

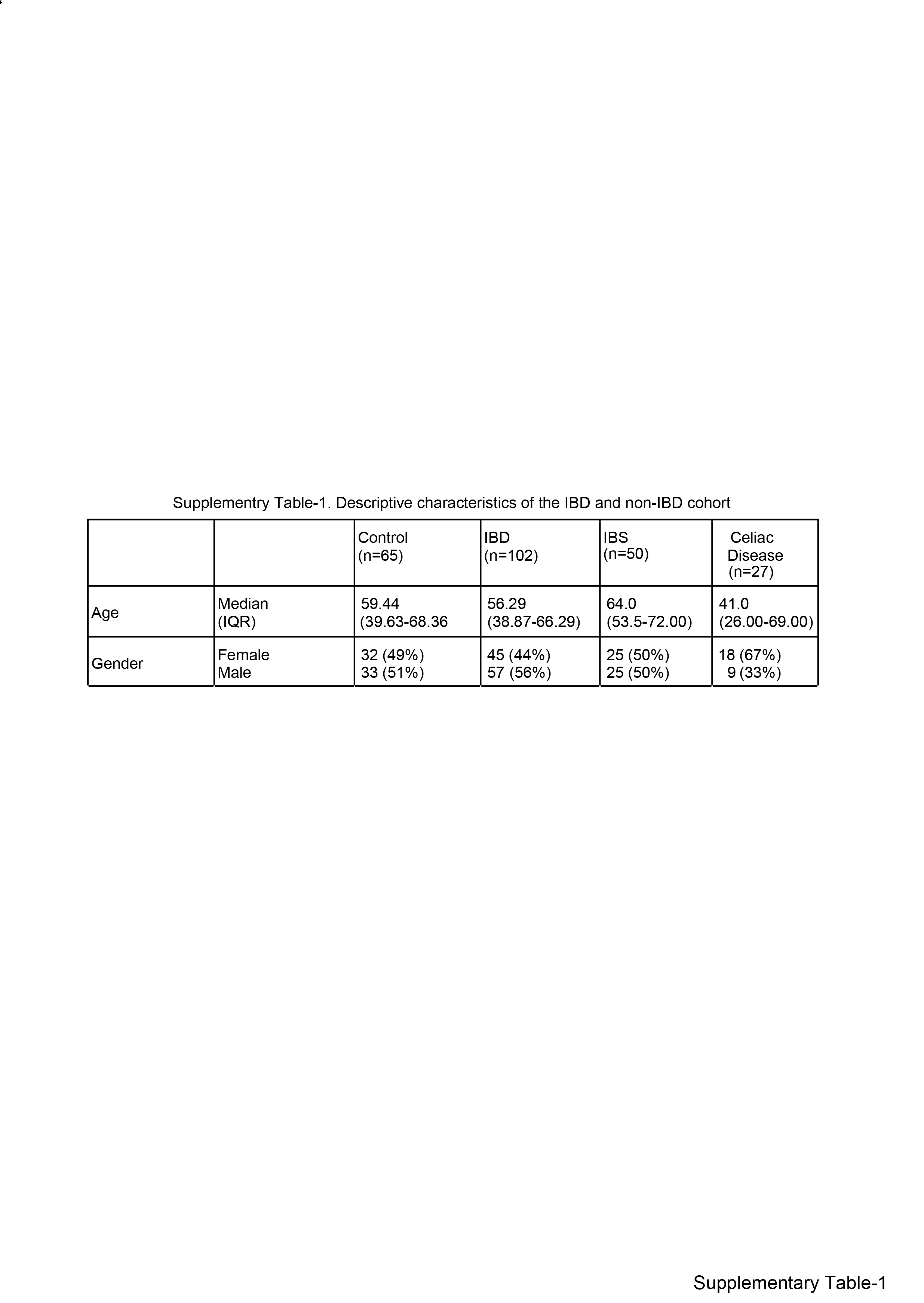

Supplement: SUPPLEMENTARY MATERIAL [file ct9-13-e00479-s001.jpg]
