# Supplementary material for: Screening Colonoscopy Findings Are Associated With Noncolorectal Cancer Mortality
Source: Clin Transl Gastroenterol. 2022 Mar 25;13(4):e00479. doi: 10.14309/ctg.0000000000000479 (PMC9038496; doi:10.14309/ctg.0000000000000479)
Supplement: SUPPLEMENTARY MATERIAL [file ct9-13-e00479-s002.docx]

Supplemental Table 1. Impact of Baseline Characteristics on All-Cause Mortality in the CSP#380 Cohort*

| **Baseline Characteristic** | **Total**  **n = 3121** | **Deaths**  **n = 858** | **Unadjusted Hazard Ratio (95% CI)** | **Adjusted Hazard Ratio^^^**  **(95% CI)** |
| --- | --- | --- | --- | --- |
| **Age, yr**  Mean (Standard Deviation, SD) | 62.92 (7.12) | 65.42 (6.4) | 1.06 (1.05-1.07) | 1.07 (1.06-1.09) |
| **Race, n (%)** |  |  |  |  |
| Caucasian | 2604 (83.4) | 714 (83.22) |  | 1 |
| African American | 297 (9.5) | 86 (10.02) | 1.08 (0.86-1.35) | 1.26 (0.98-1.61) |
| Other | 220 (7) | 58 (6.76) | 0.97 (0.74-1.27) | 1.05 (0.77-1.42) |
| **Baseline Colonoscopy Findings, n (%)** |  |  |  |  |
| No adenomas | 1950 (62.5) | 483 (56.29) |  | 1 |
| 1-2 Small adenomas | 683 (21.9) | 195 (22.73) | 1.17 (0.99-1.38) | 1.14 (0.93-1.39) |
| 3+ Small adenomas | 158 (5.1) | 61 (7.11) | 1.63 (1.25-2.13) | 1.45 (1.07-1.95) |
| Advanced adenoma | 300 (9.6) | 103 (12) | 1.49 (1.20-1.84) | 1.32 (1.00-1.75) |
| Colorectal cancer | 30 (1) | 16 (1.86) | 2.96 (1.80-4.88) | 5.65 (2.35-13.59) |
| **Baseline comorbidities, n (%)** |  |  |  |  |
| 0-2 | 2640 (84.6) | 645 (75.17) |  | 1 |
| 3-4 | 441 (14.1) | 189 (22.03) | 1.98 (1.68-2.32) | 1.56 (1.30-1.87) |
| 5+ | 40 (1.3) | 24 (2.8) | 3.37 (2.24-5.07) | 1.81 (1.07-3.07) |
| **Family History of CRC, n (%)** |  |  |  |  |
| First Degree Relative with CRC | 434 (13.9) | 97 (11.3) | 0..76 (0.61-0.94) | 0.75 (0.6-0.95) |
| **Baseline Body Mass Index** |  |  |  |  |
| 18.5-24.9 | 459 (14.8) | 157 (18.45) |  | 1 |
| <18.5 | 14 (0.5) | 8 (0.94) | 1.81 (0.89-3.70) | 1.33 (0.65-2.74) |
| 25.0-29.9 | 1359 (43.8) | 352 (41.36) | 0.70 (0.58-0.85) | 0.73 (0.60-0.90) |
| 30.0-39.9 | 1140 (36.8) | 300 (35.25) | 0.71 (0.59-0.86) | 0.75 (0.60-0.93) |
| >39.9 | 129 (4.2) | 34 (4) | 0.73 (0.50-1.06) | 1.04 (0.69-1.56) |
| **Baseline Physical Activity Index** |  |  |  |  |
| Mean score (SD) | 7.16 (1.7) | 6.81 (1.54) | 0.84 (0.80-0.88) | 0.89 (0.84-0.93) |
| **Baseline Smoking, n (%)** |  |  |  |  |
| Current daily smoker | 693 (22.2) | 259 (30.3) | 1.68 (1.45-1.94) | 2.06 (1.73-2.46) |
| **Baseline Alcohol Use** |  |  |  |  |
| Mean (SD) servings per week | 4.48 (9.61) | 4.47 (10.95) | 1.00 (0.99-1.01) | 1.00 (0.99-1.01) |
| **Baseline NSAID Use (including aspirin), n (%)** |  |  |  |  |
| None | 609 (20.1) | 155 (18.63) |  | 1 |
| Occasional | 760 (25.1) | 167 (20.07) | 0.85 (0.68-1.05) | 0.97 (0.77-1.23) |
| Daily | 1662 (54.8) | 510 (61.3) | 1.24 (1.03-1.49) | 1.19 (0.98-1.45) |
| **Baseline Beef, Pork or Lamb Consumption** |  |  |  |  |
| Mean (SD) % of total energy intake | 0.97 (0.59) | 1 (0.59) | 1.10 (0.98-1.23) | 1.06 (0.93-1.2) |
| **Baseline Cereal Fiber Consumption** |  |  |  |  |
| Mean (SD) grams | 5.69 (3.95) | 5.69 (3.9) | 1.00 (0.98-1.02) | 1.00 (0.98-1.02) |
| **Vitamin D** |  |  |  |  |
| Mean (SD) in 100 IU units | 4.28 (3.27) | 4.3 (3.49) | 1.00 (0.98-1.03) | 0.99 (0.97-1.02) |

*^*^Missing data excluded*

*^^^Adjusted for age, race, surveillance intensity and prior colonoscopy findings, number of baseline comorbidities, family history of CRC, smoking status, alcohol consumption, physical activity index, NSAIDs, body mass index, cereal fiber, red meat and vitamin D.*
